# Sidestream dark field images of the microcirculation: intra-observer reliability and correlation between two semi-quantitative methods for determining flow

**DOI:** 10.1186/1471-2342-14-14

**Published:** 2014-05-06

**Authors:** Sandra M Petersen, Gorm Greisen, Simon Hyttel-Sorensen, Gitte H Hahn

**Affiliations:** 1 Department of Neonatology, Copenhagen National Hospital, Rigshospitalet, Copenhagen DK-2100, Denmark

**Keywords:** Videomicroscopy, Microcirculation, Sidestream dark field, Total vessel density, Perfused vessel density, Microvascular flow index, Proportion of perfused vessels, Reliability

## Abstract

**Background:**

Since analysis of Sidestream Dark Field images still requires subjective interpretation, we wanted to determine intra-observer repeatability and to estimate the correlation between different evaluation methods.

**Methods:**

Fifty-four Sidestream Dark Field videos were analyzed twice by the same blinded observer using validated software. Vessels were detected, generating the parameter Total Vessel Density (TVD), and flow was determined by (i) classifying each vessel separately, generating the parameters Perfused Vessel Density (PVD) and Proportion of Perfused Vessels (PPV), and by (ii) the “Boerma” method, generating a Microvascular Flow Index (MFI) by quadrants.

**Results:**

Intraclass Correlation Coefficients (ICCs) were above 0.9 for TVD and above 0.8 for PDV and PPV. MFI_by quadrants_ had the lowest reliability (ICC = 0.52 for capillaries and ICC = 0.59 for all vessels), significantly lower than for PVD (ICC = 0.89, p < 0.001 for capillaries and ICC = 0.90, p < 0.001 for all vessels) and PPV (ICC = 0.82, p = 0.003 for capillaries and ICC = 0.83, p = 0.01 for all vessels). Correlation coefficient (r) between PPV and MFI_by quadrants_ corrected for measurement error was 0.39 (0.10 – 0.64) for capillaries and 1.01 (0.85 – 1.16) for all vessels.

**Conclusions:**

Intra-observer reliability for full evaluation of Sidestream Dark Field images was good for vessel detection and for flow classification but significantly poorer for the faster “Boerma” method. Furthermore, the Boerma method is likely to estimate different aspects of capillary flow than do the standard methods.

## Background

The microscopic techniques Orthogonal Polarization Spectral (OPS) imaging [1] and is successor Sidestream Dark Field (SDF) imaging [2] enable visualization of the microcirculation. The technique is available as a hand held device. It allows bedside investigation of the microcirculation in multiple kinds of tissue and is applied in both clinical [3,4] and experimental [5-7] research. It is argued to be the best method to evaluate heterogeneity of microcirculatory flow [8].

Software packages have been developed to evaluate SDF images semi-automatically [9]. This software detects vessels, but human interaction is still required to delete incorrectly detected vessels and to trace undetected vessels manually. Flow determination cannot yet be done automatically. It requires a semi-quantitative analysis involving subjective assessment. Two methods for flow determination are predominant: 1) the “per vessel” method, where flow in each vessel is classified separately and 2) the “Boerma” method, where the image is split into four quadrants and the predominant type of flow in each quadrant is determined.

The aim of present study was (i) to determine intra-observer agreement of vessel detection and flow classification by both the “per vessel” and the “Boerma” method; and (ii) to examine the correlation between the two methods.

## Methods

### Ethics

The animals were anaesthetized with Propofol during the entire experiment and the Danish Animal Experiments Inspectorate approved the experimental protocol (approval ID: 2009/561-1723).

### Animals

Sidestream Dark Field (SDF) videos of the cerebral cortex were recorded by one operator (G.H.H.) through a parietal craniotomy (diameter: 15 mm) in 12 newborn piglets (median age: 1 day, range: 1–6 days). Dura was removed carefully, avoiding damage to the cortex. Bleeding from the bone was avoided by Bone Wax™. During the experiment, which lasted approximately 5 hours, the cortical area was covered by wet gauzes. To prevent drying of the examined area, 2 ml saline solution (37°C) was administered every 15 minutes.

The videos were obtained as a part of a larger experimental study of cerebral blood flow published elsewhere [10]. In short the animals were exposed to blood pressure elevations of 10 mmHg by means of epinephrine, norepinephrine and dopamine. Each drug was compared to a reference method, thus giving six video sessions in each piglet.

### Sidestream dark field (SDF)

The SDF videos were obtained with a video microscope from Microscan, MicroVision Medical Inc., Amsterdam, Netherlands. The video microscope illuminates 1.08 × 0.81 mm and penetrates 0.9 mm with light in a wavelength (580 nm) that is absorbed by red blood cells (RBC). The RBCs therefore appear dark, and the viewer can trace the movement of each RBC. This gives information of the architecture of the microvasculature and erythrocyte flow in each vessel.

Image acquisition was performed according to published consensus criteria [11]. In practice, the lens was applied directly to the cerebral cortex. Adequate focus was found and in order to avoid pressure artifacts, pressure from the device was released slowly until flow could be seen in all large vessels. The videos were recorded with a length of 25 s each. Each video clip was stored as an AVI-file to allow computerized frame by frame analysis. For each session (six in total in each piglet), recordings that were out of focus or not steady enough were discarded and a new recording was made, until three videos were stored. Thus, we ended up with a total of 18 videos in each piglet.

### Experimental protocol

In the first run, the videos were given a code each, and blinded evaluation was performed on video recordings from all 12 piglets (n = 216) by one investigator (S.M.P.) who performed all steps in the analysis after the recording was made. In the second run, 3 of the 12 piglets were chosen at random, the videos (n = 54) were recoded, and blinded analysis was performed once again by the same investigator. The present study is based on the double analysis of these 54 videos.” Run 1” represents the data generated from the first evaluation and “run 2” represents the data generated from the second evaluation.

Analysis of the videos was performed using the validated software package Automated Vascular Analysis; AVA 3.0 from Micro Vision Medical Inc., Amsterdam, Netherlands [9]. A stable video fragment as long as possible was selected for further analysis, the video was corrected for background variation and the image contrast was optimized. After detecting vessels automatically, incorrectly detected vessels were deleted and undetected vessels were drawn manually or using the detection tool. Wrongly disconnected segments were chained and wrongly connected segments were unchained.

Velocity classification was performed by eye: (i) “per vessel” and (ii) with the “Boerma” method [11]. Flow was categorized as: “no flow”, “intermittent flow” (50% of the time with no flow), “sluggish flow” (slow but continuous) or “continuous flow”. To score the images with the “Boerma” method, the video was split into four quadrants, and the predominant flow of small, medium, large and very large vessels was classified in each of these quadrants. Vessels with a diameter < 20 μm was termed small [11]. In the following, these small vessels will be referred to as capillaries.

### Consensus parameters

An analysis report with the consensus parameters was created by the software program [12]. The parameter Total Vessel Density (TVD) (mm/mm^2^) reflects vessel detection. The parameters Perfused Vessel Density (PVD) (mm/mm^2^) and Proportion of Perfused Vessels (PPV) (%) reflect the “per vessel” method for evaluating flow. Perfused vessels were defined as all vessels classified as “continuous” or “sluggish” [11]. PPV was calculated by the software as the percentage of total vessel length that was perfused. The “Boerma” method for flow classification generated a Microvascular Flow Index by quadrants (MFI_by quadrants_) which was calculated by evaluating the overall flow in each quadrant (no flow = 0, intermittent = 1, sluggish = 2, continuous = 3), and then averaging the score over the four quadrants [13].

When re-analyzing the videos, the investigator started once again with the “raw” version of the video, meaning that all steps in the analysis were repeated, from choosing a stable fragment of the video to classifying the flow of the vessels.

The repeatability of SDF-video analysis was assessed by the repeatability of the consensus parameters for both capillaries (c) and for all vessels (a) as recommended [11].

### Statistics

Intra-observer repeatability was determined for each of the eight consensus parameters. The distribution of some of the parameters was not normal, thus both parametric and non-parametric methods were used. The results did not differ substantially, thus only results of parametric analysis are presented. Paired t-test was performed to compare data from run 1 and run 2. The Bland and Altman method was used to estimate agreement [14]. To assess reliability, intraclass correlation coefficients (ICCs) were calculated from a two way random effects model with absolute agreement for single measures. Since both PPV and MFI_by quadrants_ are prone to measurement error, i.e., unexplained variability, the estimated correlation coefficient between the measures will be biased towards zero. This regression dilution was corrected by the Spearman correction for attenuation [15]. Tools from http://www.vassarstats.net were used to calculate the significance of the difference between ICC’s and the confidence intervals of the correlation coefficients between PPV and MFI_by quadrants_, both with the Fischer r-to-z transformation. All other calculations were made using the program IBM SPSS statistics 20. A two tailed p-value below 0.05 was considered significant.

## Results

**Figure 1:**
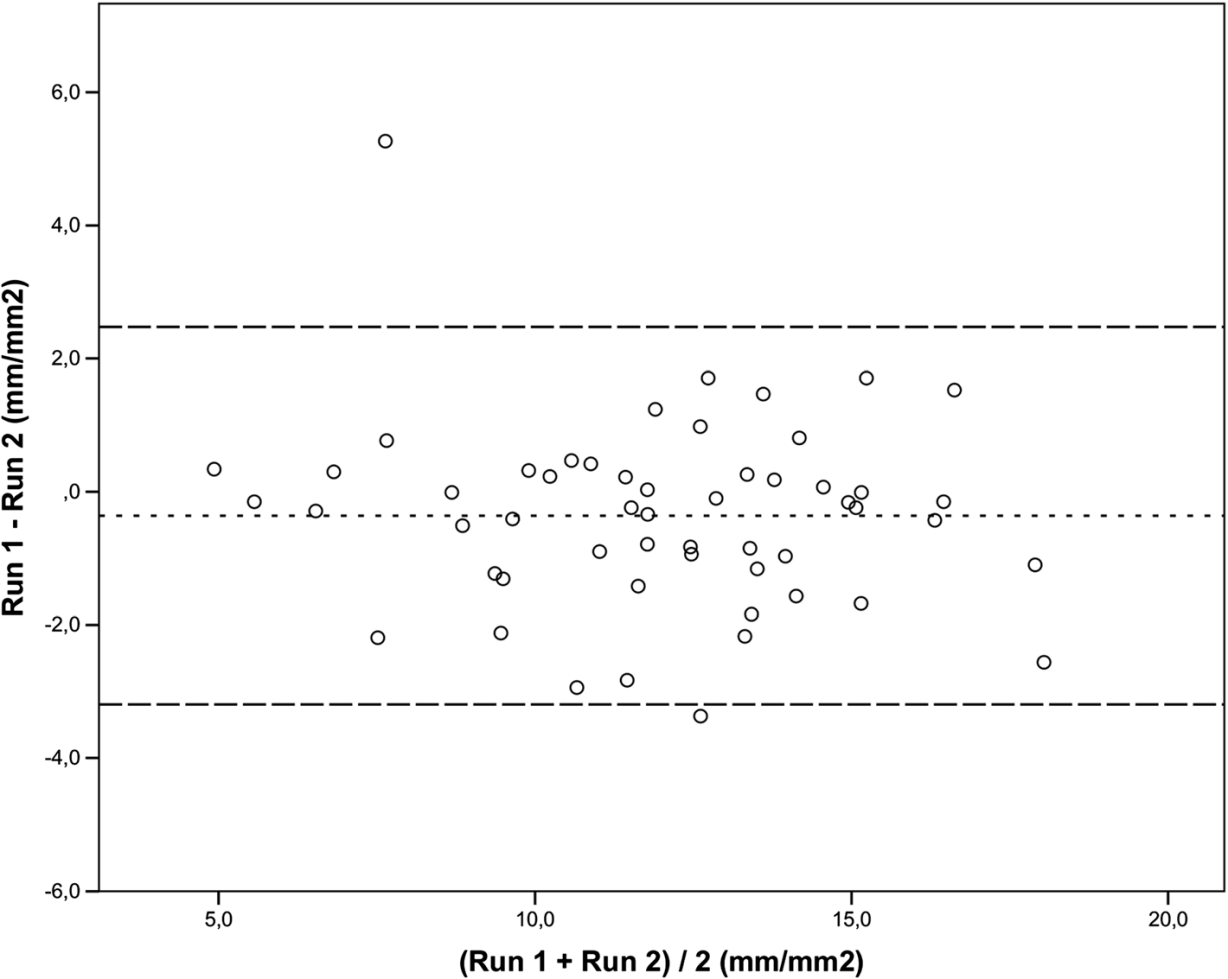
**Bland and Altman plot for capillary Perfused Vessel Density.** Figure showing bias (….) and limits of agreement (mean +/− 2 SD) (---).

All 54 videos were fully evaluated and results for the eight consensus parameters were calculated for all 108 evaluations. There was a tendency towards an increase in mean TVD and mean PVD from run 1 to run 2, but only statistically significant for TVDa (p = 0.01). PPV did not change from run 1 to run 2 whereas MFI-scoring decreased significantly from run 1 to run 2 (MFIc_by qaudrants_: p = 0.01 and MFIa_by quadrants_: p = 0.03) (Table 1). The agreement for all 8 parameters (Table 1) was inspected visually in Bland Altman plots. One outlier was seen for PVD, PPV and MFI_by quadrants_, but there was no sign of heteroscedasticity for any of the eight parameters. Perfused vessel density for capillaries is shown as an example (Figure 1).

**Table 1:** Mean and (SD), including mean difference and limits of agreement (LA)

	**Run 1 (n = 54)**	**Run 2 (n = 54)**	**p**	**Mean difference (LA)**
**Capillaries**				
TVD	13.1 (3.3)	13.4 (3.3)	0.06	−0.31 (−2.62 to 2.00)
PVD	11.8 (3.0)	12.2 (3.2)	0.07	−0.36 (−3.20 to 2.47)
PPV	65.1 (10.7)	65.8 (11.3)	0.42	−0.74 (−14.1 to 12.6)
MFI	2.7 (0.3)	2.6 (0.4)	0.01	0.12 (−0.52 to 0.77)
**All vessels**				
TVD	16.7 (2.9)	17.0 (2.9)	0.01	−0.37 (−2.53 to 1.79)
PVD	14.5 (2.9)	14.8 (3.0)	0.08	−0.31 (−2.92 to 2.29)
PPV	87.0 (8.1)	87.0 (9.8)	0.97	−0.03 (−10.6 to 10.5)
MFI	2.8 (0.2)	2.7 (0.3)	0.03	0.08 (−0.41 to 0.56)

Data is shown for Total Vessel Density (TVD, mm/mm^2^), Perfused Vessel Density (PVD, mm/mm^2^), Proportion of Perfused Vessels (PPV, %) and Microvascular Flow Index (MFI).

The ICC was higher than 0.5 for all 8 parameters (Table 2). TVD, the parameters reflecting vessel detection, had the highest reliability with an ICC of 0.94 for capillaries and 0.92 for all vessels. Regarding flow classification, MFI_by quadrants_ had the lowest reliability (ICC = 0.52 for capillaries and ICC = 0.59 for all vessels), significantly lower than for PVD (ICC = 0.89, p < 0.001 for capillaries and ICC = 0.90, p < 0.001 for all vessels) and PPV (ICC = 0.82, p = 0.003 for capillaries and ICC = 0.83, p = 0.01 for all vessels).

**Table 2:** Intraclass Correlation Coefficient (ICC) and 95% Confidence interval (CI)

	**ICC (95% CI)**
**Capillaries**	
TVD	0.94 (0.89 – 0.96)
PVD	0.89 (0.82 – 0.94)
PPV	0.82 (0.70 – 0.90)
MFI_by quadrants_	0.52 (0.29 – 0.70)
**All vessels**	
TVD	0.92 (0.86 – 0.98)
PVD	0.90 (0.83 – 0.97)
PPV	0.83 (0.72 – 0.90)
MFI_by quadrants_	0.59 (0.38 – 0.74)

Data is shown for Total Vessel density (TVD), Perfused Vessel Density (PVD), Proportion of Perfused Vessels (PPV) and Microvascular Flow Index by quadrants (MFI_by quadrants_).

**Figure 2:**
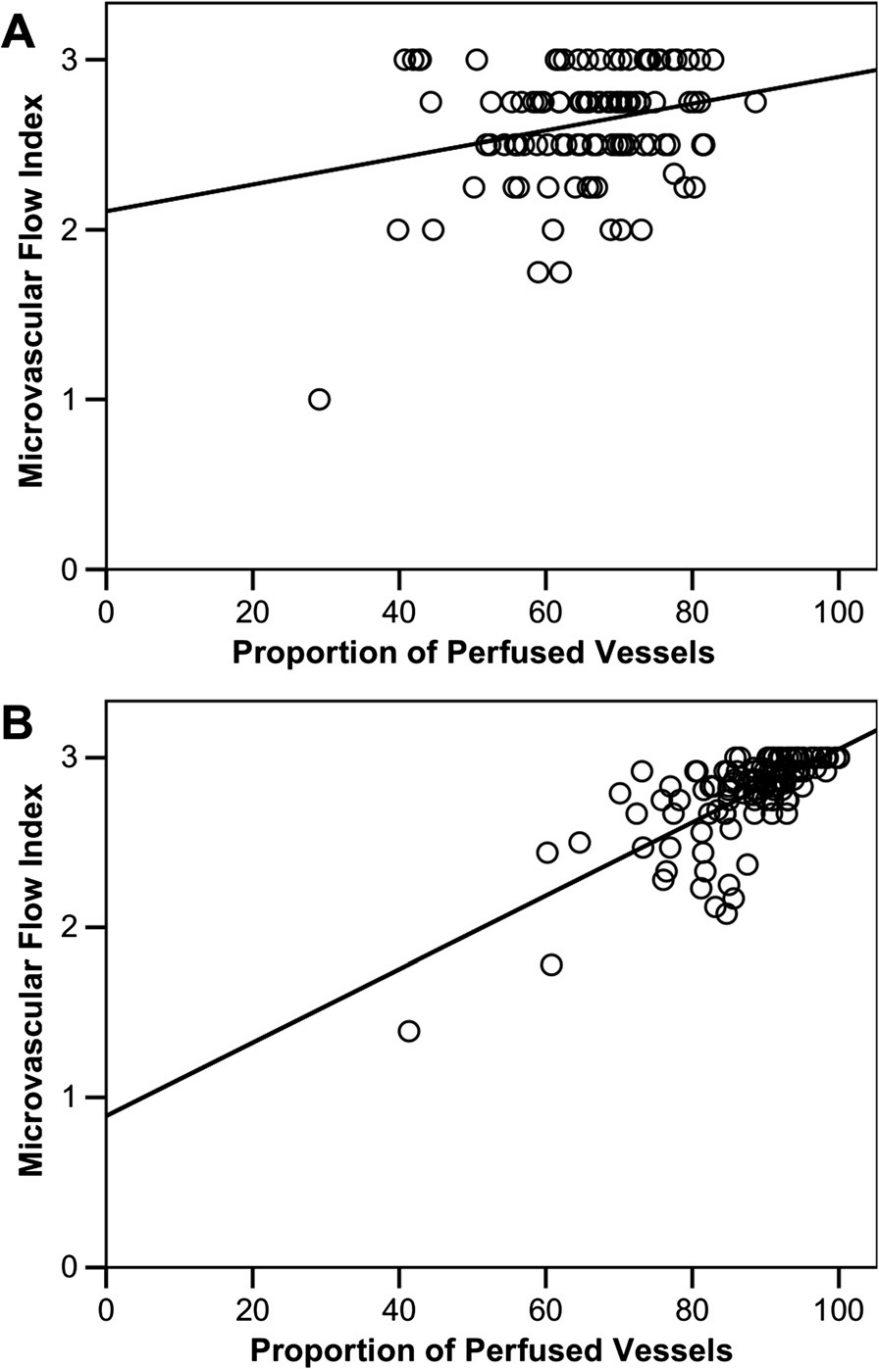
**Correlation between Proportion of Perfused Vessels and Microvascular Flow Index by quadrants.** Correlation is shown for capillaries **(A)** and for all vessels **(B)**.

The correlation between MFI_by quadrants_ and PPV was visualized in scatter plots (Figure 2). Correlation coefficient (r) between PPV and MFI_by quadrants_ was 0.25 (0.066 – 0.42) for capillaries and 0.71 (0.60 – 0.81) for all vessels. After the Spearman correction for attenuation the correlation coefficient was 0.39 (0.10 – 0.64) for capillaries and 1.01 (0.85 – 1.16) for all vessels.

## Discussion

This blinded intra-observer study shows that the repeatability for semi-quantitative analysis of SDF imaging is good for both vessel detection and flow classification with reliability above 0.9 for vessel detection (TVD) and above 0.8 for flow evaluation using the “per vessel” method (PVD and PPV). For the “Boerma” method (MFI_by quadrants_), however, reliability was relatively weak. Furthermore, the correlation between PPV and MFI_by quadrants_ was low for capillaries but almost perfect for all vessels after correcting for the measurement error found in this study.

The strengths of this study are: (i) We studied anaesthetized piglets, and the only movement artifact will be that of the investigator holding the camera which is a realistic setting for most animal studies. (ii) Image acquisition was performed as recommended in a published round table report to minimize movement artifacts, pressure artifacts and focus problems [11]. (iii) The investigator evaluating the videos was not involved in the process of acquiring images and the evaluations were performed blinded keeping bias at a minimum. (iv) An instruction was made before analyzing the videos to make sure that each video was evaluated in a standardized manner. This instruction was based on the manual and on experiences from an evaluation performed on a random video by two of the investigators (S.M.P. and G.H.H.). Finally, (v) the investigator analyzed a total of 216 videos before reanalyzing 54 of them again for this study, making it impossible to remember the videos from run 1 when presented for them again in run 2. The small drift in the mean value of some of the parameters from run 1 to run 2 shows that the standardization was effective although not perfect.

This study has limitations as well: (i) The investigated tissue was cerebral cortex depleted of dura and cerebrospinal fluid for several hours, thus the visualized microcirculation and quality of the images might have been affected. (ii) Image quality might also have been affected by the experimental setup, where the piglets were exposed to several induced blood-pressure elevations. (iii) Finally, due to movement artifacts the stabilized videos became shorter than 20 seconds in some of the videos, and in these cases classification of vessels had to be based on shorter fragments than recommended.

We reported a low reliability of the MFI_by quadrants_. In another study, the “Boerma” method was validated using kappa statistics, and high kappa values for both intra- and inter-observer variability were reported (kappa = 0.75 and 0.85 respectively) [13]. In our study, the mean MFI_by quadrants_ was high (2.6 – 2.8), because most of the quadrants were given the classification 3 for continuous flow (Figure 2), whereas in the validation study the classification of quadrants was more equally distributed between 0 and 3, and a mean of 2.2 for sublingual site classifications and 1.8 for stoma site classifications could be calculated from the published data. This will of course influence the reliability of the method, since videos with high between-subject variability are easier to distinguish than videos with less variability. Apart from that it might reflect that it is easier to distinguish between intermittent and sluggish flow (1 and 2) than between sluggish and continuous flow (2 and 3).

Recently, the “Boerma” method was compared to a method where every vessel was classified, and a parameter called MFI_vessel by vessel_ was generated as the mean of all the classifications [16]. SDF images were evaluated with both methods, and the agreement between them was poor (bias +/− precision 0.21 ± 0.73, *P* = 0.05). The MFI_vessel by vessel_ was better correlated to RBC velocity (calculated using space time diagrams) and to the PPV, than the MFI_by quadrants_. This supports the findings of our study suggesting that a more reliable evaluation of the images requires classification of each vessel separately.

In our study, we calculated the correlation between PPV and MFI_by quadrants_. Low correlation was found for capillaries, even after correction for measurement error. A low correlation was also found between PPV and MFI_by quadrants_ (R^2^ = 0.54, P = 0.0001) in the study concerned with different kinds of MFI’s [16], but in a recent study of patients with sepsis a stronger correlation for capillaries was found (R^2^ = 0.92, p = 0.0001) [17]. However, none of the studies corrected for measurement error. Both studies used videos obtained from critically ill patients, and MFIs were more equally distributed between values of 0 and 3 than in our study. Based on these correlations it cannot be concluded whether or not PPV and MFI evaluate capillary flow in the same way. Furthermore, we found a correlation above 1 for all vessels. This might indicate an underestimate of the reliability of the methods [18].

The “Boerma” method has some general problems apart from the lack of reliability. It does not differ between very heterogeneous flow and generally homogenous, but sluggish flow. As an example, if flow is very heterogeneous, two quadrants might be classified as having continuous flow, and the other two as having intermittent flow (3, 3, 1 and 1). This yields an MFI_by quadrants_ of 2, as would also be generated if all four quadrants were classified as having sluggish flow (2, 2, 2 and 2). Thus, two very different situations will be interpreted as the same using the MFI_by quadrants_. Furthermore, MFI is a categorical parameter and an increase from 1 to 2 does not necessarily imply the same amount of change in perfusion as an increase from 2 to 3. Further studies are needed to establish if this could be part of the explanation of the low correlation found between PPV and MFI_by quadrants_ for capillaries in our study. The high correlation for all vessels might be explained by larger vessels being easier to classify than the small capillaries.

The “Boerma” method also has some advantages compared to the “per vessel” method. Classifying each vessel separately is a time-consuming process and evaluating a video file of 20 seconds duration with the “per vessel” method takes between 10 and 45 minutes depending on number of capillaries in the area, the contrast of the images, movement-artifacts and bleeding in the tissue. With a modification of the “Boerma” method a real-time, point of care evaluation can be made in two minutes [19]. New rapid automated methods for vessel density assessment has been developed but automated velocity assessment still has many limitations, especially if heterogeneity is to be assessed [20,21]. If SDF imaging is used in research, an evaluation time of approximately 30 minutes per video will be acceptable, but if it is supposed to become a clinical tool, evaluation time has to be short. This represents the main advantage for the” Boerma” method.

The remaining question is, if the parameters generated from the analysis of SDF images provide information that can’t be found using less time consuming techniques. As laser Doppler technique can provide information of changes in blood flow [22], bedside assessment of heterogeneity remains the most promising new aspect of SDF. Clinical studies using SDF have demonstrated increased microcirculatory heterogeneity in patients with sepsis compared to healthy volunteers, and larger changes among non-survivors than survivors [17,23]. As described earlier, the MFI does not provide information regarding heterogeneity, neither does PPV. A heterogeneity index has been proposed where images recorded from three to five sites are analyzed, and highest minus lowest site MFI divided by mean MFI of the sites are calculated [23]. Low reliability of MFI and the natural oscillations of the vasculature over time [24] taken into account, this method may not be able to detect small changes in heterogeneity. In that respect PPV seems as the superior method for evaluation of microcirculatory heterogeneity.

Does the quality of image acquisition affect the analysis of the images? In our study one major outlier serves as an example of how image acquisition problems can affect later evaluation. The outlier was detected for all velocity parameters when inspecting the Bland Altman plots (Figure 1). The video-file was inspected and revealed a constant slight movement of the camera in the 20 second long sequence. Comparison of the two different stabilized video-files generated from run 1 and 2 showed that two different parts of the video was selected for stabilization. This means that the rest of the analysis was made on two different parts of the video. Therefore video recordings with good quality are essential. The hand held device might not enable such high quality recordings [25]. An image acquisition stabilizer has been developed, but is not available for clinical use [26].

## Conclusions

In conclusion, this study demonstrated that intra-observer repeatability for evaluation of SDF images was good for vessel detection (TVD) and for flow classification, when each vessel was classified separately (PVD and PPV). A significantly poorer repeatability was found for the “Boerma” method and PPV therefore seems as the superior method for evaluation of microcirculatory heterogeneity, especially when detecting small changes. Furthermore, correlation between the MFI_by quadrants_ and PPV after correcting for measurement error was low for capillaries but almost perfect for all vessels. Thus, it seems that the Boerma method quantifies different aspects of capillary flow than does PPV.

## Abbreviations

SDF: Sidestream dark field; TVD: Total vessel density; PVD: Perfused vessel density; PPV: Proportion of perfused vessels; MFI: Microvascular flow index; c: For capillaries; a: For all vessels; ICC: Intraclass Correlation Coefficient.

## Competing interests

All authors declare that they have no competing interests.

## Authors’ contributions

SMP made all analyses of the SDF images, conducted the statistical analyses and drafted the manuscript. GHH and SHS conducted the piglet studies and GHH recorded the SDF images. GG supervised the statistical analysis. GG and GHH conceived of this study and supervised the drafting of the manuscript. All authors revised the manuscript for important intellectual content and approved the final draft.

## Pre-publication history

The pre-publication history for this paper can be accessed here:

http://www.biomedcentral.com/1471-2342/14/14/prepub
